# The nature and nurture of mental health problems in the family

**DOI:** 10.1371/journal.pmed.1004867

**Published:** 2026-01-06

**Authors:** Jasmin Wertz, Leah S. Richmond-Rakerd

**Affiliations:** 1 Department of Psychology, University of Edinburgh, Edinburgh, United Kingdom; 2 Department of Psychology, University of Michigan, Ann Arbor, Michigan, United States of America

## Abstract

Mental health problems are known to run in families, but it is not clear to what extent this reflects nature or nurture. In this Perspective, Jasmin Wertz and Leah Richmond-Rakerd highlight a recent PLOS Medicine article that disentangles these influences to shed light on how mental health problems are transmitted across generations.

## Teaser

Mental health problems are known to run in families, but it is not clear to what extent this reflects nature or nurture. By disentangling these influences, a recent *PLOS Medicine* article sheds light on how mental health problems are transmitted across generations.

Mental health problems are well known to cluster in families, an observation that has long captured researchers’ attention. For example, the risk of experiencing depression is approximately two to three times higher for children of depressed parents compared to children of nondepressed parents [1,2]. A key question about this familial clustering is to what extent it reflects nature or nurture: Do parents with mental health problems pass on genetic vulnerability to their children, or does parental mental illness affect the family environment in ways that influence children’s own mental health? If transmission is entirely due to environmental influences, then addressing parental mental illness and its effects on the family environment would be sufficient to disrupt the intergenerational transmission of mental health problems. If transmission is entirely due to genetic influences, then changes to parental mental health and the family environment—although still beneficial, including for children at genetic risk of mental health problems [3]—would not in itself be enough to disrupt transmission.

Disentangling environmental and genetic pathways of transmission is challenging because it requires a comparison of children who are matched in genetic risk but differ in exposure to parental mental health problems. A valuable research design that approaches this natural experiment is the children-of-twins design. Children of identical twins are as genetically similar to their own parent as they are to their aunt or uncle, but their environmental exposure to mental health problems may differ. This design therefore addresses the hypothetical of how a child’s development may have differed had they (not) grown up with a parent experiencing mental illness.

In a recent *PLOS Medicine* study [4], Zhou and colleagues used this design to test associations between parental psychiatric diagnoses and offspring outcomes, primarily mental health outcomes, but also behavioral and psychosocial outcomes such as convictions for violent crimes and educational attainment. The authors’ findings confirm that these associations are largely due to genetic transmission from parent to child. However, they also find evidence for environmentally-mediated effects of parental mental illness on offspring outcomes; in particular, on offspring risk for mental health problems. For example, children whose parent had internalizing mental health problems (e.g., depression) had a 26% higher risk of developing a mental health problem themselves, compared to their cousin whose parent did not have internalizing mental health problems. Notably, these environmental associations were transdiagnostic: Children exposed to both parental internalizing conditions (e.g., depression) and externalizing conditions (e.g., substance use disorder) were more likely to experience any type of psychiatric condition themselves. This mirrors findings for genetic risk factors for mental health problems, which also cross diagnostic boundaries.

Methodologically, this study showcases the value of administrative data, which are generated through people’s interactions with administrative systems (e.g., healthcare, social-welfare, education, and criminal-justice systems). In Sweden, administrative data are available at the population level, which made it possible to use a design relying on identical twin births, which are relatively rare (approximately three to four per 1,000 births). The very large scale of these data also made it possible to link family members across generations and observe how their lives unfold over long follow-up periods—up to 51 years in this study [4]—which is challenging in survey studies. Furthermore, the linkage of administrative databases across domains enabled the authors to uncover associations of parental mental health problems with not only offspring mental health outcomes, but also crime, education, and labor-market outcomes. For example, they find that parental internalizing problems were not only associated with an increased risk of mental health problems in children, but also with lower educational attainment, highlighting how mental health problems co-occur with social problems not only within, but also across generations.

For mental health research and treatment, these findings highlight the importance of an intergenerational perspective, particularly the need for a greater family focus in mental health services. Mental health support for adults tends to be geared toward the individual and does not necessarily record whether the patient is a parent. Even in child and adolescent mental health services, research suggests that parental mental illness is not consistently assessed or addressed [5]. This represents a missed opportunity in several ways. First, mental illness within families often involves bidirectional pathways, with parent and child mental health problems influencing and exacerbating one another as well as shaping overall family functioning. A focus on the whole family, in addition to individual support, can help to address such bidirectional influences and contribute to improved family climate [6]. Second, research suggests that information about family history of mental illness has prognostic value for predicting clinical features of young people’s mental health problems, such as risk of disorder recurrence and degree of impairment [7]. In addition, the presence of parental mental illness can affect young people’s treatment response and adherence and therefore has implications for treatment planning [5]. Third, given that mental health problems tend to be underdiagnosed and undertreated [8], a greater consideration of the familial aggregation of mental health problems could guide targeted screening efforts to identify people at the highest risk for developing mental health problems and make support available in a timely manner. A parallel is the approach of cascade screening for some medical conditions with a substantial familial component, where a diagnosis in one individual triggers proactive screening of their family members [9]. Recent research shows that a screening approach based on transdiagnostic familial psychiatric risk could identify a quarter of bipolar disorder cases in the population [10].

Another aspect these intergenerational relationships highlight is the need to support an individual’s mental health earlier, before they become a parent. Mental health problems, particularly those that are severe and recurrent and thus most likely to be transmitted across generations, often onset in adolescence or early adulthood, typically before the transition to parenthood [11]. This creates an opportunity for intervention, both by investing in mental health services for young people, as well as by better supporting prospective parents during the preconception and perinatal period. Many parents-to-be are more engaged with healthcare services during this time and may be particularly receptive to messaging about health support and promotion. It is also a period when parents with existing mental health problems are more vulnerable to relapse [12] and may experience fears about passing on their mental health problems to their children [13]. In recent years, many healthcare systems have increased screening for perinatal mental health problems and improved perinatal support for women with a history of mental health problems. For example, specialist community perinatal mental health teams were rolled out in England from 2016, with their availability shown to improve access to care and reduce risk of relapse (although effects on birth outcomes were more mixed) [14]. Through supporting (prospective) parents with mental health problems, such initiatives have the potential to proactively address the environmental transmission pathways of mental health problems, thereby promoting the well-being of the next generation.

Taken together, the findings from this study [4] show that genetic influences are an important reason for why mental health problems are transmitted from parents to children, but that environmental factors also matter. Through early identification of individuals at greatest familial risk and proactive support for parents with mental health problems, a family focus in mental health services can address both inherited vulnerabilities and modifiable environmental pathways to improve mental health across generations.
